# Posttraumatic Corpus Luteal Cyst Rupture: A Diagnostic Enigma for Massive Hemoperitoneum

**DOI:** 10.7759/cureus.37067

**Published:** 2023-04-03

**Authors:** Richa Yadav, Mayoukh Sarkar, Juniad Alam, Dinesh Bagaria

**Affiliations:** 1 Radiology, All India Institute of Medical Sciences, New Delhi, New Delhi, IND; 2 Surgery, All India Institute of Medical Sciences, New Delhi, New Delhi, IND; 3 Trauma and Acute Care Surgery, All India Institute of Medical Sciences, New Delhi, New Delhi, IND

**Keywords:** hemorrhage, trauma, ct, fast, corpus luteum cyst

## Abstract

In the case of trauma, the presence of free fluid in the abdominal cavity on the focused assessment with sonography for trauma scan usually indicates the possibility of hemoperitoneum caused by injury to the abdominal organs. However, on rare occasions, isolated injuries to gynecologic organs can also result in hemoperitoneum, especially among women of reproductive age. Thus, the rupture of a corpus luteal cyst may be one of the myriad causes of massive hemoperitoneum and carries a risk of misdiagnosis for patients with trauma.

In this case report, we highlight the characteristic imaging findings of a case of apoplexy of the corpus luteum cyst that presented to the emergency department as a cause of massive hemoperitoneum after blunt abdominal trauma.

## Introduction

Hemoperitoneum can occur in various emergency scenarios. In the case of trauma, the presence of free fluid in the abdominal cavity on the focused assessment with sonography for trauma (FAST) scan usually indicates the possibility of hemoperitoneum due to injury to the abdominal organs. In decreasing order of frequency, the most commonly injured abdominal organs following blunt abdominal trauma are the spleen, liver, kidney, small bowel, and mesentery [1,2]. On rare occasions, isolated injuries to gynecologic organs can also result in hemoperitoneum.

Therefore, a radiologist should always be alert for common and rare causes of hemoperitoneum. We present a case of blunt abdominal trauma that led to the rupture of an incidental corpus luteal cyst as a culprit of the massive hemoperitoneum. We highlight the salient CT imaging features of a ruptured corpus luteal cyst to enable an efficient diagnosis.

## Case presentation

A 26-year-old female presented with trauma after an alleged fall from a height that directly impacted her lower abdomen. The primary survey revealed that her vitals were within the normal range, but mild tachycardia was recorded (102 beats/minute). Bruising was conspicuous over the lower abdomen. Per the institutional trauma protocol, the eFAST revealed the presence of free fluid in all four quadrants, including the epigastrium, hepatorenal, perisplenic, and pelvic regions. The patient’s pneumo-scan and urine pregnancy test (UPT) results were negative. Afterward, per our institutional protocol, she underwent a non-contrast CT (NCCT) scan of the head and cervical spine and a contrast-enhanced CT (CECT) scan of the torso with both arterial and venous phases.

**Figure 1:**
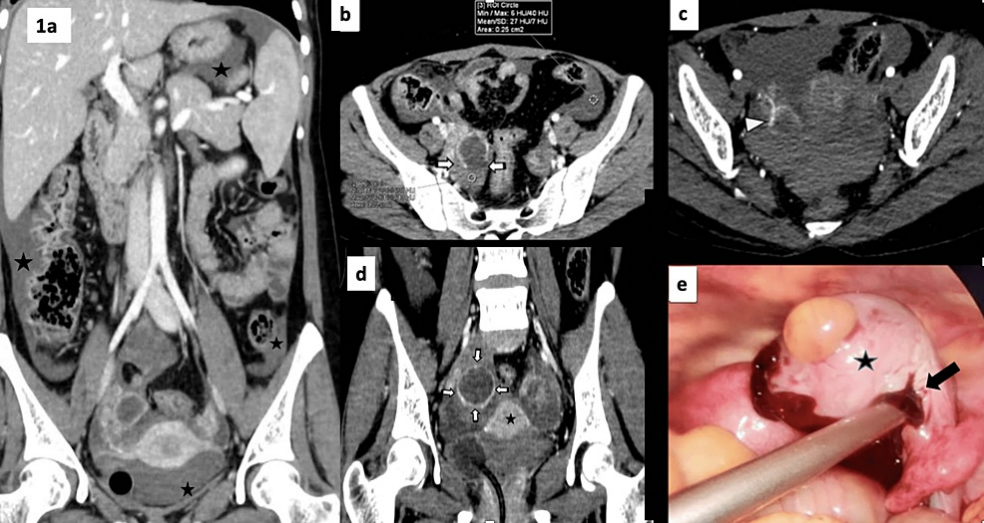
CT and diagnostic laparoscopy images. Contrast-enhanced CT of the lower abdomen showing gross hemoperitoneum in all four quadrants around perihepatic, perisplenic, epigastrium, and pelvic regions in coronal reformated images (a). On axial sections (b, c), a rim-enhancing hypodense cystic lesion was seen in the right adnexa with a focal discontinuity in the posterior wall at 4 to 6 o’clock positions (white arrows, b), indicating a ruptured cyst (direct sign) with high attenuation fluid (HU ~51), suggesting a sentinel clot near the ruptured site representing acute clotted hemorrhage (white arrowhead, b, c). Coronal reformatted images showing irregular thick wall enhancement (d) and CT “ring of fire appearance,” suggesting impending rupture or a tensile cyst (arrow, d). Myometrial enhancement was used as a reference (d, star). Diagnostic laparoscopy images (e) showing a large corpus luteal cyst (star) with a cauterized rent and a drain placed (arrow).

The CECT of the torso revealed the presence of high-attenuation free fluid (>30 HU) in all four quadrants around the perihepatic, perisplenic, epigastrium, and pelvic regions (Figure 1, Panel a), which was suggestive of gross hemoperitoneum. No obvious solid organ, bowel, or mesenteric injury was observed. In the pelvis, the right ovary was found to be bulky, and a well-defined hypodense cyst measuring approximately 3 cm with a thick, enhancing wall was observed within (Figure 1, Panels b-d). The cyst had crenulated margins in places, with focal discontinuity in the wall noted in the posterior-medial aspect (Figure 1, Panels b, c). The free fluid adjacent to the cyst was found to be of higher attenuation values (>45 HU) (Figure 1, Panels b-d). The following two possibilities were considered: a ruptured ovarian cyst, most likely corpus luteum, or an occult bowel or mesenteric injury. Owing to a negative UPT and normal beta-human chorionic gonadotropin (β-HCG) level, the possibility of ruptured ectopic pregnancy was ruled out.

Outcome and follow-up

The patient underwent diagnostic laparoscopy (DL) in view of persistent tachycardia and the presence of gross hemoperitoneum. Intraoperatively, gross hemoperitoneum was observed around the right ovary as well in all four quadrants. Further, a large cyst was confirmed in the right ovary with an irregular crenated surface and a small rent, which was cauterized (Figure 1, Panel e). The hollow viscus organs and mesentery were unremarkable. The postoperative course was unremarkable.

## Discussion

The corpus luteal cyst is a functional vascular ovarian structure with tiny, fragile vessels in the wall that are prone to rupture, resulting in intracystic hemorrhage, intraperitoneal rupture, and massive hemoperitoneum [3]. Its manifestations vary from asymptomatic to symptomatic, mimicking the symptoms of acute abdomen, secondary to peritoneal irritation due to prostaglandin release after cyst rupture.

The etiology of the corpus luteal cyst varies from spontaneous rupture to lower abdominal trauma. Its recognized risk factors include anticoagulation therapy, strenuous physical activity, and iatrogenic factors such as uterine examination [4]. As was witnessed in this case, pelvic trauma is one of the rare etiologic factors. Its plausible mechanisms are direct compression force or acceleration-deceleration injury to the lower abdomen. The existing literature has noted that the right ovary is more susceptible to rupture than the left because differential ovarian venous outflow results in increased intraluminal pressure on the right side, and the ovary on the left side is protected by the cushioning effect of the rectosigmoid colon [5].

Imaging is essential in diagnosis and characterization and helps in timely management. Ultrasonography (USG) is an easily accessible, non-invasive, real-time, and radiation-free method. Hence, this method is preferred for the initial evaluation and diagnosis of simple and complicated corpus luteal cysts effectively only in routine or follow-up cases, but in level 1 trauma centers where eFAST is performed as an adjunct to the primary survey to observe the presence of free fluid (i.e., bleeding) in the peritoneal and pleural cavity and not to look actively for pelvic organ. Therefore the major findings of a complicated corpus luteal cyst on USG can be easily missed.

MRI is the investigation of choice for better characterization of adnexal lesions in routine or follow-up cases but not in acute trauma settings, where CECT of the torso is the tool of choice to identify the source of hemoperitoneum after a positive FAST status. The most common cause of hemoperitoneum is solid organ injury, followed by bowel and mesenteric injuries and, on rare occasions, gynecologic injuries, as was observed in the present case.

On CECT, the corpus luteal cyst appears as a hypodense adnexal cystic structure with a thick, enhancing wall [3]. Direct or indirect features associated with an impending or ruptured corpus luteal cyst become evident on the CT scan. The indirect CT signs are the “ring of fire” sign, the sentinel clot sign, the irregular shape, and the presence of massive hemoperitoneum [6]. In contrast, the direct CT signs are focal discontinuities in the wall or incomplete “ring of fire” signs and active contrast extravasation, which are potential indicators for surgical treatment [7]. An irregular wavy border represents the post-rupture collapse of the cyst. The “ring of fire” sign, originally described in color Doppler ultrasound findings of a corpus luteum cyst as increased cyst wall flow, is seen as a more prominent enhancement of the potentially involved cyst wall with a visual comparison of the myometrium as an internal reference [8]. This sign represents a tensile cyst and indicates impending or contained rupture (Figure 1d). Further, the sentinel clot sign is seen as focal, high-density clotted blood around the ovarian cyst on the CECT images and represents acute clotted hemorrhage around the cyst (Figures 1b-1d).

The differential diagnosis in the case of hemoperitoneum includes ruptured ectopic pregnancy, which a negative pregnancy test and the serum β-HCG level can be used to rule out. Another differential diagnosis is the spontaneous rupture of a corpus luteum cyst, which is more common in the luteal phase of the menstrual cycle during pregnancy or after infertility treatment. The absence of pain in the abdomen before the trauma, a bruise over the lower abdomen on clinical examination, and fresh blood during surgery favor posttraumatic rupture over spontaneous rupture.

The management strategy is either conservative or surgical, depending on the clinical and imaging presentation. Unstable vital signs, uncontrolled pain with massive bleeding, and active contrast extravasation on CECT are indications of immediate surgical intervention [4]. In this case, DL was performed in view of the gross hemoperitoneum and persistent tachycardia.

## Conclusions

Causes of rupture of corpus luteum cysts vary from spontaneous to traumatic. Although traumatic rupture of an incidental corpus luteal cyst is rare, it should always be included in the differential diagnosis of women of childbearing age who present with a large hemoperitoneum after trauma without injury to a solid organ. The plausible mechanism could be a direct impact or deceleration injury in the lower abdomen. Because of the difference in anatomic embryology, the right ovary is more vulnerable than the left. Therefore, CECT of the abdomen is an important tool in the trauma setting for localizing the anatomic site of hemorrhage (sentinel clot sign) and effectively visualizing the direct or indirect signs of a complicated luteal cyst as described with varying sensitivity.
